# Modulating the effective ionic radii of trivalent dopants in ceria using a combination of dopants to improve catalytic efficiency for the oxygen evolution reaction†

**DOI:** 10.1039/d4ra03360d

**Published:** 2024-06-03

**Authors:** Debarati Das, Jyoti Prakash, Anisha Bandyopadhyay, Annu Balhara, U. K. Goutam, Raghunath Acharya, Santosh K. Gupta, Kathi Sudarshan

**Affiliations:** a Radiochemistry Division, Bhabha Atomic Research Centre Mumbai-400085 India kathis@barc.gov.in santoshg@barc.gov.in; b Homi Bhabha National Institute Anushaktinagar Mumbai – 400094 India; c Materials Group, Bhabha Atomic Research Centre Mumbai-400085 India; d Radiation and Photochemistry Division, Bhabha Atomic Research Centre Mumbai-400085 India; e Technical Physics Division, Bhabha Atomic Research Centre Mumbai-400085 India

## Abstract

Aliovalent doping in ceria and defect engineering are important aspects in tuning the properties of ceria for advanced technological applications, especially in the emerging field of electrocatalytic water-splitting for harvesting renewable energy. However, the ambiguity regarding the choice of dopants/co-dopants and ways to deal with the size difference between dopants and lattice hosts remains a long-standing problem. In this study, ceria was aliovalently codoped with Sc^3+^ and La^3+^ while keeping the total concentration of dopants constant; the ionic radius of the former is smaller and that of the latter is larger than Ce^4+^. Variations in the relative amounts of these dopants helped to modulate the effective ionic radii and match that of the host. A systematic study on the role of these aliovalent dopants in defect evolution in ceria and in modulating the Ce^3+^ fraction using powder XRD, Rietveld refinement, positron annihilation lifetime spectroscopy, X-ray photoelectron spectroscopy, Eu^3+^ photoluminescence, and Raman spectroscopy is presented here. The evolved defects and their dependence on subtle factors other than charge compensation are further correlated with their electrocatalytic activity towards oxygen evolution reaction (OER) in alkaline medium. The catalyst with an optimum defect density, maximum Ce^3+^ fraction at the surface and the least effective ionic radius difference between the dopants and the host demonstrated the best performance towards the OER. This study demonstrates how effective ionic radius modulation in defect-engineered ceria through a judicious choice of codopants can enhance the catalytic property of ceria and provides immensely helpful information for designing ceria-based heterogeneous catalysts with desired functionalities.

## Abstract 2

Matching effective ionic radii of trivalent dopants with the host through judicious choice of dopants and codopants has emerged as a smarter strategy for improving OER catalytic activity of ceria than individual ion doping.

## Graphical abstract

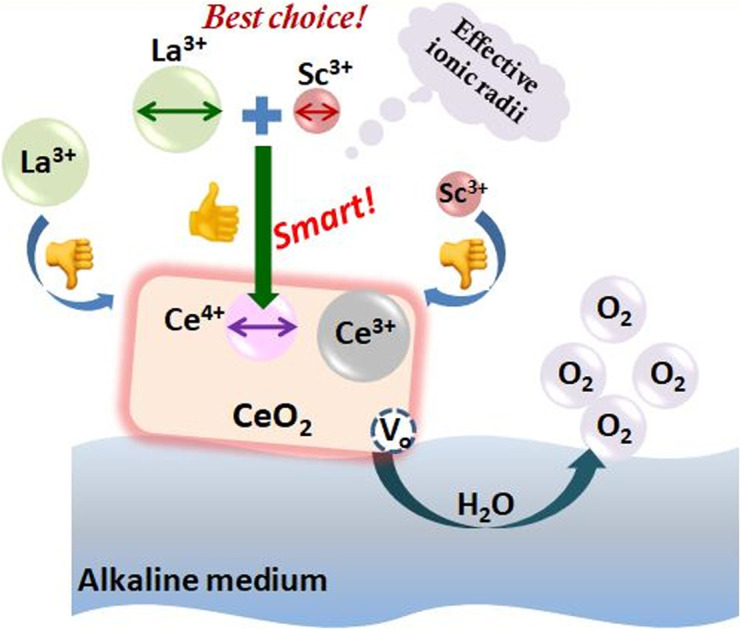

## 1. Introduction

Defects are known to play a pivotal role in determining the optical, electrical, catalytic, magnetic and various other properties of importance to the technological applications of advanced functional materials. Accordingly, engineering defects to tailor or improve the properties of interest has gained prominence.^1–3^ Ceria (CeO_2_) is one such system on which numerous studies have been carried out to explore its potential in diverse technological applications such as redox catalysis, photocatalysis, organic transformation, gas sensing and thermal barrier coatings owing to its remarkable structural stability, superior oxygen storage capability, surface activity and transport properties.^2,4,5^ In the last several decades, water-splitting reactions, *viz.* the oxygen evolution reaction (OER) and hydrogen evolution reaction (HER), using electrocatalysis have acquired great importance in the fabrication of renewable energy devices such as rechargeable batteries, supercapacitors and regenerative solid-oxide fuel cells for sustainable energy applications.^6–8^ Being a low-cost and earth-abundant rare-earth oxide with outstanding mechanical stability and exceptionally high corrosion resistance, ceria has been used as a catalyst for water splitting.^9–11^ Aliovalent doping is a well-known strategy to introduce desired defects in ceria by exploiting charge and size mismatch between the host and dopant ions to accelerate its catalytic response.^11–13^ Doping not only impacts the active sites of adsorption such as oxygen vacancies but also promotes the ion-transport mechanism through vacancies and the readily switchable Ce^3+^/Ce^4+^ redox pair. However, tailoring defects in ceria to improve catalytic efficiency depends on the judicious choice of aliovalent dopants that particularly promote water-splitting reactions. Trivalent lanthanide dopant incorporated ceria has been investigated for enhanced water-splitting activity for solar thermochemical hydrogen generation,^13–15^ electrochemical water splitting^16,17^ and other catalytic reactions.^18,19^ Despite many studies on defect evolution in ceria with different dopants for various technological applications, a clear picture is still to emerge regarding various factors that dictate the overall properties of the doped ceria. Therefore, it is imperative to gain a further understanding of the various factors influencing electrocatalytic efficiency and draw a systematic correlation between the dopants and desired outcomes.

In the simplest case, doping of two trivalent ions at the tetravalent Ce cation site would lead to an oxygen vacancy but the easy reducibility of Ce^4+^ to Ce^3+^, differences in the ionic radii of the dopants with respect to the host lattice and the relative distributions of oxygen vacancies and dopants, as well as their mutual association in the matrix, make the situation more complex. For example, some studies have reported increased Ce^3+^ concentration and oxygen vacancy concentration with smaller trivalent ion doping^20^ due to their ability to accommodate larger Ce^3+^ without much lattice distortion; other groups have shown lower Ce^3+^ concentrations with smaller dopants because their strong association with oxygen vacancies does not aid the reduction of Ce^4+^ to Ce^3+^.^17,21^ Vinothkumar *et al.*^19^ reported an enhancement in oxygen vacancy (V_O_) concentration with increasing ionic radii of the lanthanide dopants; however, Ke *et al.*^22^ suggested an initial increase in oxygen vacancy concentration with the size of the trivalent lanthanide dopant followed by a reduction with larger lanthanides. A decrease in Ce^3+^ concentration at higher La doping levels is also reported and is attributed to the change in the defect structure from La^3+^-V_O_-Ce^3+^ to La^3+^-V_O_-La^3+^ with increased doping.^23^

The studies so far show the complexities and intricacies in understanding the factors influencing the evolution of defects in trivalent-ion-doped ceria. This study is aimed at gaining a better understanding of the factors that influence the defect evolution other than the concentration of trivalent dopants and their role in governing its reactivity towards electrocatalytic water splitting. For this purpose, ceria was doped with Sc^3+^ and La^3+^, which have ionic radii smaller and larger than Ce^4+^, respectively, to modulate the effective ionic radii of the trivalent dopants and understand their influence on OER reaction catalysis. The total concentration of the trivalent dopant was kept constant at 20 mole% with respect to Ce to unravel subtle factors governing defect evolution, their distribution, and Ce^3+^ content other than charge compensation. The ceria catalysts were synthesized by a gel-combustion method, and doping was confirmed by powder X-ray Diffraction (XRD), Rietveld refinement and neutron activation analysis. The crystallite size and strain were further determined using the Williamson–Hall method, and the morphology was investigated using Transmission-Electron Microscopy. The evolution of oxygen vacancies was examined using Positron Annihilation Lifetime Spectroscopy (PALS) and Raman spectroscopy. The surface oxygen vacancy concentration and Ce^3+^ fraction were determined using X-ray photoelectron spectroscopy (XPS). Photoluminescence (PL) was employed to extract information about the distribution of oxygen vacancies using the hypersensitivity of electric dipole transitions in Eu^3+^ manifesting the features of the local surroundings. The ceria catalysts were explored for OER in alkaline media, and their catalytic activity was further correlated with effective ionic radii modulation, evolved defects, lattice strain and reducibility to Ce^3+^. The findings from this study will be extremely useful in further designing ceria-based composite catalysts with enhanced activity of the ceria counterpart.

## 2. Experimental methods

### 2.1. Synthesis of powder ceria catalysts

All the samples were synthesized by the gel-combustion method reported in our earlier studies using urea as the fuel.^17,24^ Cerium nitrate hexahydrate (Ce (NO_3_)_3_·6H_2_O) and urea were used in a 1 : 2 molar ratio as the starting materials. La_2_O_3_, Sc_2_O_3_ and Eu_2_O_3_ were dissolved individually in concentrated nitric acid to prepare stock solutions. Appropriate volumes of these dopant solutions were added to the aqueous solutions of precursors so that the total concentration of Sc^3+^ and La^3+^ was 20 mole% of cerium nitrate. The concentration of Eu^3+^ was kept fixed at 4 mol% of Ce in all the doped samples. The solution was heated until it converted to a gel under an IR lamp and then heated in a tubular furnace initially for a few minutes at 250 °C for the combustion of urea, which was followed by crushing, further heating for three hours at 500 °C and finally, annealing at 800 °C for four hours. Ambient air flow was used in the tubular furnace during heat treatment.

### 2.2. Structural, morphological and compositional characterization of as-synthesized ceria

The calcined ceria powders were characterised by the powder XRD technique using a benchtop XRD (Proto Make) in the 2*θ* range of 20 to 80 degrees at 2 degrees per minute. The experimentally acquired XRD patterns were further subjected to Rietveld refinement using FullProf software, and a Williamson–Hall analysis was carried out to calculate lattice strain and crystallite size from the XRD pattern. The morphology of a representative codoped ceria catalyst was investigated by employing High-Resolution Transmission Emission Microscopy (HRTEM) using a FEI Talos F200S instrument at 200 kV. To determine the dopant concentration, Neutron Activation Analysis (NAA) was carried out with approximately 10–15 mg of each sample along with standards made up of homogeneous mixtures of CeO_2_ and the individual dopant oxides, namely La_2_O_3_, Sc_2_O_3_ and Eu_2_O_3_. The samples along with the standards were irradiated in the ‘self-serve’ irradiation facility of Dhruva research reactor, Bhabha Atomic Research Centre, Mumbai for two hours. The irradiated samples were assayed by gamma spectrometry with a 30% relative efficiency HPGe detector with an energy resolution of 2 keV at 1333 keV of ^60^Co.^25^ The concentration of the dopants was estimated based on the specific activity of the standard oxides irradiated under the same condition.

### 2.3. Defect spectroscopy of the ceria catalysts

To understand defect evolution in the ceria catalysts, Positron Annihilation Lifetime Spectroscopic (PALS) studies were carried out using a fast–fast coincidence positron lifetime spectrometer equipped with two fast-timing BaF_2_ scintillation detectors with an overall time resolution of ∼173 ps. Carrier-free ^22^Na (∼15 μCi), which was used as the positron source, was immersed in a sufficient quantity of the powder samples in a sandwich configuration, and acquisition was done for ∼1 million counts. The experimental lifetime spectra were convoluted using the instrumental resolution function and fitted using the PALSFit3 software to extract the different lifetime components.^26^ The PAL spectrum of Si was used as the reference to evaluate the fraction of positron annihilating within the source. To further obtain insights into the surface distribution of defects and Ce^3+^, XPS measurements were carried out at the synchrotron radiation facility using hard X-ray photoelectron spectroscopy (HAXPES) beamline BL-14, Indus-2 (ref. 27) equipped with a double-crystal monochromator [Si (111)] with an excitation energy of 4.359 keV and a hemispherical analyzer and detector system (Phoibos 225, Specs make). The typical pressure in the experimental station was maintained at 5 × 10^−9^ mbar.

Photoluminescence (PL) measurements were carried out on an FLS1000 spectrometer (Edinburgh Instruments) equipped with 450 W steady-state xenon lamps. Raman spectra were recorded on a home-built Raman spectrometer that utilized a continuous-wave 532 nm Nd:YAG laser of 50 mW power, and further details of instrumentation are given elsewhere.^28^

### 2.4. Electrochemical measurements

To evaluate the catalytic activity of the ceria catalysts towards OER, electrochemical studies were performed in N_2_-saturated 1 M KOH solution (pH 14) using a CHI760 galvanostat/potentiostat electrochemical workstation. A glassy carbon electrode (3 mm diameter) was used as the working electrode, while an Ag/AgCl (3 M KCl) electrode and Pt wire were used as the reference and counter electrodes, respectively, in the three-electrode set-up. The catalyst ink was prepared by dispersing 5 mg of each catalyst in a mixed solution of 250 μL of iso-propanol, 750 μL of water and 50 μL of 5 wt% Nafion, which acted as the binder. The catalyst ink was homogenized by ultrasonication for 30–45 minutes, and 10 μL (5 μL at a time) of the ink was drop-casted over the clean surface of the GCE with a geometrical area of 0.0706 cm^2^. The electrode was kept within a closed enclosure for 45 minutes for drying. To evaluate the OER activity, Linear Sweep Voltammograms (LSV) were recorded in the potential range of 0 to 1.5 V *vs.* Ag/AgCl at a scan rate of 2 mV s^−1^. Impedance measurements were carried in the frequency range of 1 Hz to 100 kHz at a fixed potential of +1.0 V *vs.* Ag/AgCl electrode. The potentials were converted with respect to the reversible hydrogen electrode using the following equation.

1 *E*(RHE) = *E*(Ag/AgCl) + *E*_0_(Ag/AgCl) + 0.0591pH

The EIS data were analysed using the EC Lab software to model the equivalent circuit and extract the charge transfer resistance.^29^ Chronopotentiometric measurement was performed with the best-performing catalyst to check the stability of the catalyst.

## 3. Results and discussion

### 3.1. Powder X-ray diffraction

**Fig. 1:**
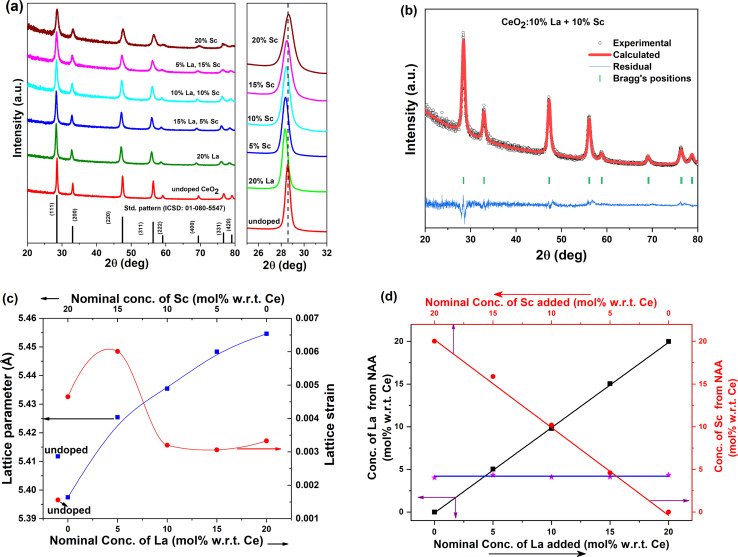
(a) Powder XRD patterns of Sc^3+^ and La^3+^ codoped CeO_2_ samples. (b) A typical Rietveld-refined XRD pattern. (c) Variations in the lattice parameter and lattice strain upon codoping (the lines shown are eye-guides only). (d) Dopant concentrations in the doped ceria samples determined using neutron activation analysis.

The powder XRD patterns of all the samples prepared are given in Fig. 1a. The standard pattern of CeO_2_ (ICSD no. 01-080-5547) is also shown at the bottom. The XRD patterns of all the samples match quite well with the standard pattern of CeO_2_ corresponding to the fluorite structure with the space group *Fm*3̄*m*. No additional unidentified peaks were observed, indicating the phase purity of the samples, and the shifts in peak positions further assure the incorporation of dopants in the lattice. The ionic radii of La^3+^, Sc^3+^, Ce^3+^ and Ce^4+^ in 8 coordination were 1.16, 0.87, 1.14, and 0.97 Å respectively. It was also noted that the XRD peaks of the La-rich sample were shifted to lower angles compared with the undoped sample due to the larger size of the La^3+^ dopant than Ce^4+^. Similarly, the XRD patterns of the Sc-rich samples were shifted to higher angles due to the smaller ionic radii of Sc^3+^, and both these suggest the incorporation of dopants in the lattice of Ce^4+^.

#### 3.1.1. Rietveld refinement and Williamson–Hall analysis

To further quantify the changes in lattice parameters upon doping and codoping, Rietveld refinement analysis of the experimentally acquired XRD patterns was carried out. The typical fitting of a codoped sample is shown in Fig. 1b, and the fitted patterns along with the fitting parameters are shown in Fig. S1 and Table S1 in the ESI† file. It is evident from the variation in the lattice parameter depicted in Fig. 1c that the lattice parameter of 20% Sc-doped CeO_2_ was less than that of undoped ceria (5.4117 Å); in all other cases, the lattice parameter increased upon doping. The enhancement observed is the manifestation of the synergistic effect of the aliovalent dopant size, lattice relaxation and the alteration in Ce^3+^ content. The largest cell parameter was observed when the larger dopant La was doped at the maximum concentration. The weighted average of the Shannon radii of the dopants present in ceria is shown in Fig. S2.† The average radius of dopants in the 15% Sc + 5% La-doped ceria sample was the closest to that of the host. As discussed later, this sample also showed better OER efficacy and higher Ce^3+^ concentrations.

To determine the lattice strain and crystallite size of the ceria samples, Williamson–Hall analysis was carried out with the experimentally acquired patterns. The details of the methodology are elaborated in the ESI along with the calculated crystallite size in Table S1.† The crystallite sizes of the doped catalysts were found to be lower than that of undoped ceria. This is quite similar to our previous finding that trivalent ion doping restricts crystallite growth. The variation in lattice strain is demonstrated in Fig. 1c. The maximum lattice strain was observed in the case of 15% Sc + 5% La codoped CeO_2_. The strain decreased with the gradual incorporation of the larger dopant La^3+^, possibly due to lattice expansion, and the strain was marginally higher in the 20% La-doped sample.

### 3.2. Neutron activation analysis and morphological studies

To further validate the presence of dopants in ceria, the neutron-irradiated samples were assayed using gamma-ray spectroscopy. The typical gamma-ray spectrum from a codoped sample is shown in Fig. S3.† The gamma rays corresponding to each of the dopants could be identified in the spectrum. The measured peak areas (PA) were corrected for decay during counting and cooling time to get the relative activity (not corrected for detector efficiency) at the time of the end of irradiation (*A*_EoI_), as shown below:2
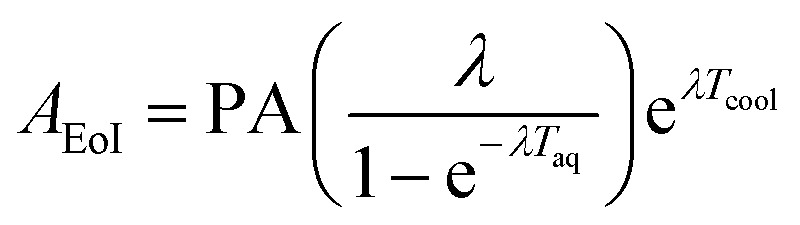
where *λ* is the radioactive decay constant of the nuclei being monitored, *T*_aq_ is the acquisition duration of the gamma spectrum, *T*_cool_ is the time elapsed between the end of irradiation and the start of the gamma-spectrometric assay. The mass of the element of interest in the samples (*m*_samp_) was calculated from relative activity at the end of irradiation as follows.3
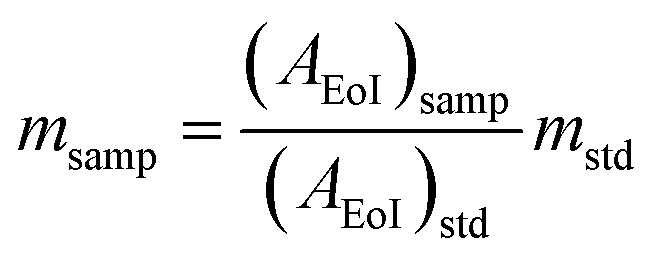
where *m*_std_ is the mass of the element of interest in the standard. The mass of the element was converted to mole% of dopant with respect to Ce. The dopant concentrations determined by neutron activation were plotted against the nominal concentrations used during synthesis, as shown in Fig. 1d. It can be seen from the figure that the determined dopant concentrations match well with the nominal concentrations taken. The elemental composition along with the Rietveld-refined powder XRD patterns suggest the quantitative incorporation of dopants in the lattice at the concentrations used during synthesis in the present study.

**Fig. 2:**
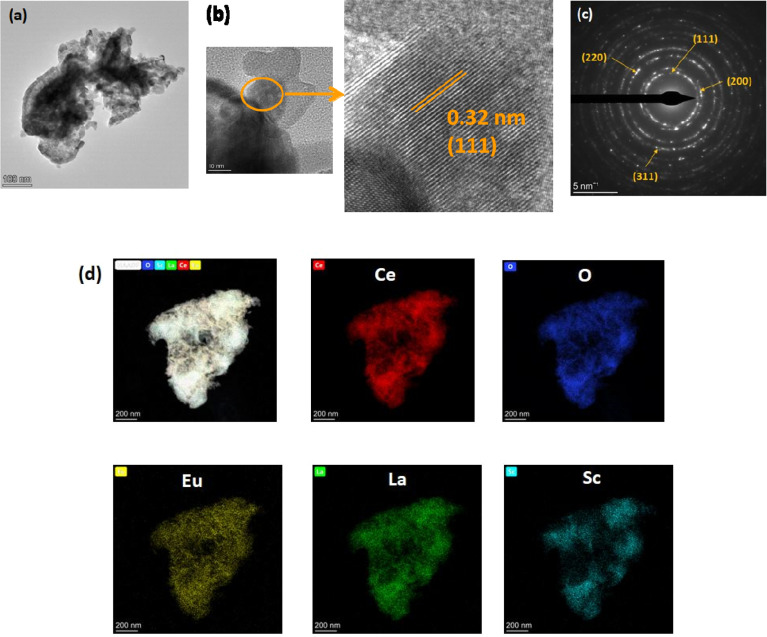
(a and b) TEM images at two different magnifications. (c) SAED pattern and (d) STEM-HAADF elemental mapping of 15% Sc and 5% La codoped CeO_2_ nanoparticles.

The TEM micrographs of the 15% Sc + 5% La codoped CeO_2_ nanoparticles at two different magnifications are shown in Fig. 2a and b, and the selected area electron diffraction pattern is shown in Fig. 2c. The micrograph in Fig. 2a shows agglomeration of the smaller nanoparticles, which is frequently observed in the case of ceramic nanopowders synthesized *via* a sol–gel or gel-combustion route. The uniform elemental distribution of the dopants in the ceria matrix was further confirmed by the High-Angle Annular Dark-Field (HAADF) images of the ceria nanoparticles, as depicted in Fig. 2d.

### 3.3. Positron annihilation lifetime spectroscopy

The PAL spectra of the undoped and doped CeO_2_ catalysts were fitted as the sum of three lifetime components, of which the third component (*τ*_3_) in the range of 1–2 ns was attributed to *o*-Ps pick-off annihilation at the surface and inter-grain boundaries. This *τ*_3_ does not bear much relevance in our present study, and therefore, subsequent discussions are centred around the first two lifetime components and their corresponding intensities only. A typical fitting of the lifetime spectrum is shown in Fig. S4.†

**Fig. 3:**
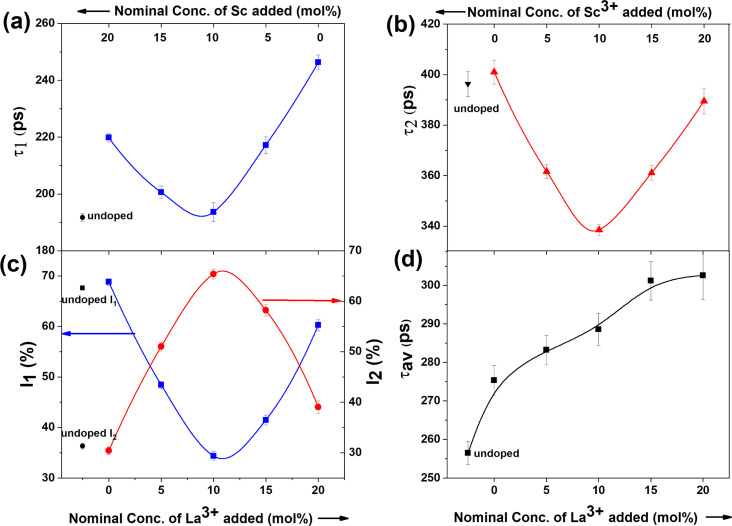
(a and b) Variations in the first and second positron lifetime components; (c) their corresponding intensities and (d) intensity-weighted average positron lifetimes of the ceria catalysts. The lines in the figures are guides to the eye.

The first lifetime component (*τ*_1_) of undoped ceria was obtained as 192 ps, which is slightly higher than the previously reported experimental bulk lifetime of 184 ps, as well as the theoretically calculated lifetime of 172 ps.^30^ This enhancement in *τ*_1_ can be attributed to the presence of inherent oxygen vacancies that as shallow positron traps in ceria, quite similar to our previous works.^24,31^ As evident from Fig. 3a, *τ*_1_ increased upon doping, indicating the creation of a greater number of oxygen vacancies. With an increase in the relative concentration of La^3+^ (or decrease in Sc^3+^), both *τ*_1_ and *I*_1_ initially decreased (Fig. 3a and c) and showed minima around CeO_2_:10% Sc, 10% La and later increased. *τ*_1_ is usually ascribed to positron annihilation from the delocalised bulk, and its lifetime is indistinguishable from the lifetime of positrons from shallow positron traps like oxygen vacancies. The variations in *τ*_1_ and *I*_1_ suggest that the smaller oxygen vacancies, such as monovacancy and neutral Ce^3+^-V_O_ or Ln^3+^-V_O_ vacancy, reduce initially with an increase in the La^3+^ dopant fraction. The second lifetime component *τ*_2_ is ascribed to the presence of oxygen vacancy aggregates that act as large vacancy clusters near the surface. This lifetime also showed minima with maxima in its corresponding intensity when both Sc^3+^ and La^3+^ were doped equally (Fig. 3b and c). The positron data together suggest that the vacancy aggregates grow in number initially and in cluster size at higher La concentrations. The smaller crystallite size of the codoped catalysts could be another auxiliary factor responsible for high *I*_2_, thereby allowing a greater fraction of positrons to diffuse to the surface of the crystallites. The average positron lifetime, which was calculated as the intensity-weighted average of the two positron lifetimes (Fig. 3d), increased monotonically with the increase in La^3+^ fraction, showing an overall increase in defect concentration with increasing La fraction, which is in agreement with previous studies.^17,19,22^ The growth of vacancy clusters near the surface, as shown by an increase in *τ*_2_, might be responsible for the reduction in lattice strain despite the La^3+^ dopant being larger in size than Ce^4+^. The weaker association of charge-compensating vacancies with the dopant in the case of large La dopant as compared to in the case of a high Sc content might be responsible for the formation of larger vacancy clusters in the former.^32,33^

### 3.4. X-ray photoelectron spectroscopy (XPS)

To extract information about the Ce^3+^ fraction and changes in oxygen vacancy concentration at the surface upon doping, XPS measurements were carried out. The survey scans did not show any impurity peaks for the samples.

**Fig. 4:**
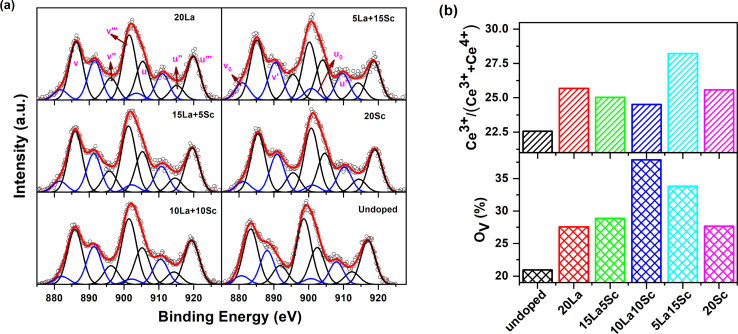
(a) Fitted Ce-3d XPS spectra of the ceria catalysts; (b) Ce^3+^ and oxygen vacancy concentrations on the surface of the ceria catalysts, as calculated from the Ce-3d and O-1s XPS spectra, respectively.

Due to the coexistence of both Ce^3+^ and Ce^4+^ and their possible mixtures in the final state configurations, the XPS spectrum from cerium in ceria appeared to be quite complex. The Ce-3d XPS spectra could be deconvoluted to ten peaks due to the splitting originating from spin–orbit coupling, giving rise to the Ce 3d_5/2_ and Ce 3d_3/2_ regions, and each further consisted of five peaks due to the presence of Ce^3+^, Ce^4+^ and other possible configurations.^34^ Because of the intrinsic complexity of the spectra and to minimize the number of variables to be fitted, the widths of the corresponding peaks in the Ce 3d_5/2_ and Ce 3d_3/2_ regions were taken equal, and the relative intensity was also fixed as 3 : 2. The spectra showed six peaks located at 885.9 eV (*ν*), 896.2 eV (*ν*′′), 901.5 eV (*ν*′′′), 905.3 eV (*u*), 914.3 (*u*′′) and 919.3 (*u*′′′) related to Ce^4+^ and four components at 880.9 eV (*ν*_0_), 890.4 eV (*ν*′), 900.8 eV (*u*_0_) and 909.9 eV (*u*′) corresponding to the main and satellite peaks of Ce^3+^. The nomenclature of the peaks is based on the literature, and the positions of the peaks are in good agreement with the previously reported results.^35^ The typical XPS spectra from Ce-3d fitted to different components are shown in Fig. 4a. The fraction of Ce^3+^ on the surface was determined as the ratio of the total fractional area under the peaks *ν*_0_, *u*_0_, *ν*′ and *u*′ to the total area of all the peaks given above. The percentage of Ce^3+^ on the surface of the samples thus evaluated is given in Fig. 4b.

Similarly, the O-1s XPS spectra (as shown in Fig. S5, ESI†) could be deconvoluted into three components located at binding energies ∼529.8 eV, ∼533.1 eV and ∼535.6 eV. Based on the literature, the peak at 529.8 eV is attributed to lattice oxygen bound to Ce^4+^ (O_L_), while the peak at 533.1 eV corresponds to oxygen bound to Ce^3+^ or the signature of oxygen vacancies (O_v_) and the third peak at 535.6 eV arises due to adsorbed oxygen (O_A_).^36^ The fractional areas under the peaks at ∼533.1 eV were evaluated as the relative oxygen vacancy concentrations on the surface of ceria and are given in Fig. 4b. As evident from Fig. S5,† there was a shift in the peak maximum of the peak corresponding to O_v_ and the intensity of both ∼533.1 and ∼535.6 eV peaks were higher in the doped samples compared with undoped ceria.

It can be seen from Fig. 4b that undoped ceria showed a Ce^3+^ fraction of about 22.5%, which increased with trivalent ion doping in all cases. Moreover, the Ce^3+^ concentration was higher when ceria was codoped with 15% Sc and 5% La than in the case of single doping with either 20% Sc or 20% La.

On the other hand, the surface oxygen vacancy concentration was also observed to be higher in all the doped samples than in undoped ceria, for which the O_v_ concentration (under coordinated oxygen) was calculated as 20.95%. The variation in oxygen vacancy concentration at the surface is also quite similar to the trend of the intensity (*I*_2_) variation of the second positron lifetime component pertaining to surface defects. In other words, it can be suggested that the abundance of oxygen vacancy clusters is the highest at the surface when the two codopants are doped in an equal stoichiometric ratio.

Some studies in the literature have reported that the proportion of trivalent cerium positively correlates with the concentration of oxygen vacancies.^37,38^ In the studies referred to above, CeO_2_ was singly doped with one particular heteroatom from the same row of transition metals at a time, and they were lower in ionic size than that of Ce. Therefore, it can be suggested that, in this study, the simultaneous doping of multiple trivalent ions with appreciable differences in ionic radii with respect to Ce might lead to the evolution of oxygen vacancies to varied extents. The oxygen vacancies also have varied tendencies of association with different-sized dopants besides forming vacancy clusters among themselves. Therefore, identifying a one-to-one correlation between O_v_ and trivalent Ce is hard in this case. A similar scenario has also been manifested in our previous study on a similar ceria system.^17^

### 3.5. Time-resolved photoluminescence

**Fig. 5:**
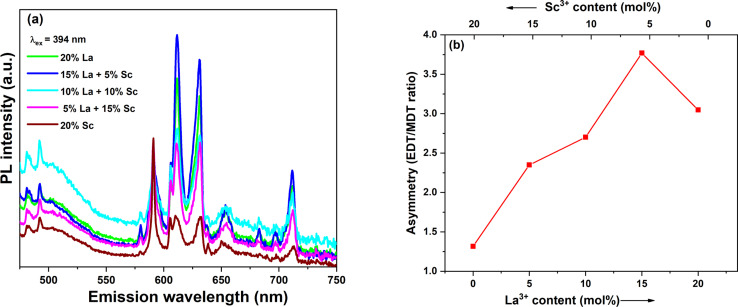
(a) Emission spectra with *λ*_ex_ at 394 nm, and (b) asymmetry ratios calculated from Eu^3+^ emissions of the trivalent-ion-doped samples.

As discussed earlier, all the samples were spiked with Eu^3+^ in order to use the luminescence emission from Eu^3+^ to probe the local structure around the dopants. It is well-known that the emission from Eu^3+^ is highly sensitive to its surroundings due to the presence of hypersensitive electric dipole transitions (EDT). The photoluminescence emission spectra of all the doped samples were recorded in the region corresponding to Eu^3+^ emission with excitation at 394 nm and are shown in Fig. 5a. The prominent transitions are marked in the spectra. The most intense transitions found in the spectra are ^5^D_0_ → ^7^F_1_ and ^5^D_0_ → ^7^F_2_. It is well-known that the magnetic dipole transition (MDT) ^5^D_0_ → ^7^F_1_ is dominant when Eu^3+^ is present at a site with inversion symmetry, while the electric dipole transition (EDT) ^5^D_0_ → ^7^F_2_ is dominant when Eu^3+^ is present at a site with non-inversion symmetry. The Ce^4+^ site in CeO_2_ is of cubic symmetry with an inversion centre. To understand the symmetry around Eu^3+^, asymmetry in the emission signal was calculated as the ratio of areas under the emission peaks corresponding to EDT/MDT. The calculated asymmetry ratios are shown in Fig. 5b. The asymmetry ratio initially increased with the La^3+^ fraction and reached the maximum for the 15% La + 5% Sc codoped sample. An increase in asymmetry with the variation in trivalent dopant concentration in ceria is well-known due to the increase in oxygen vacancy concentration.^31,39,40^ However, it may be noted that the trivalent ion content in all the samples studied here was constant, and only the relative fraction of Sc^3+^ (smaller trivalent) to La^3+^ (larger trivalent) was changed. Based on the charge balance, the oxygen vacancy concentration should remain constant. The increase in asymmetry with an increase in La^3+^ or lowering of asymmetry at high Sc^3+^ fractions shows that Eu^3+^ is in a more symmetric environment when the Sc^3+^ fraction is high. This may be attributed to the strong association of oxygen vacancies with smaller trivalent ions, which enhances the symmetry around Eu^3+^ due to the removal of oxygen vacancies, as reported by various theoretical studies.^33,41^ Thus, in Sc^3+^-rich doped samples, the dominant vacancies seem to be Sc^3+^-V_O_-Sc^3+^ and Sc^3+^-V_O_-Ce^4+^.

### 3.6. Raman spectroscopy

**Fig. 6:**
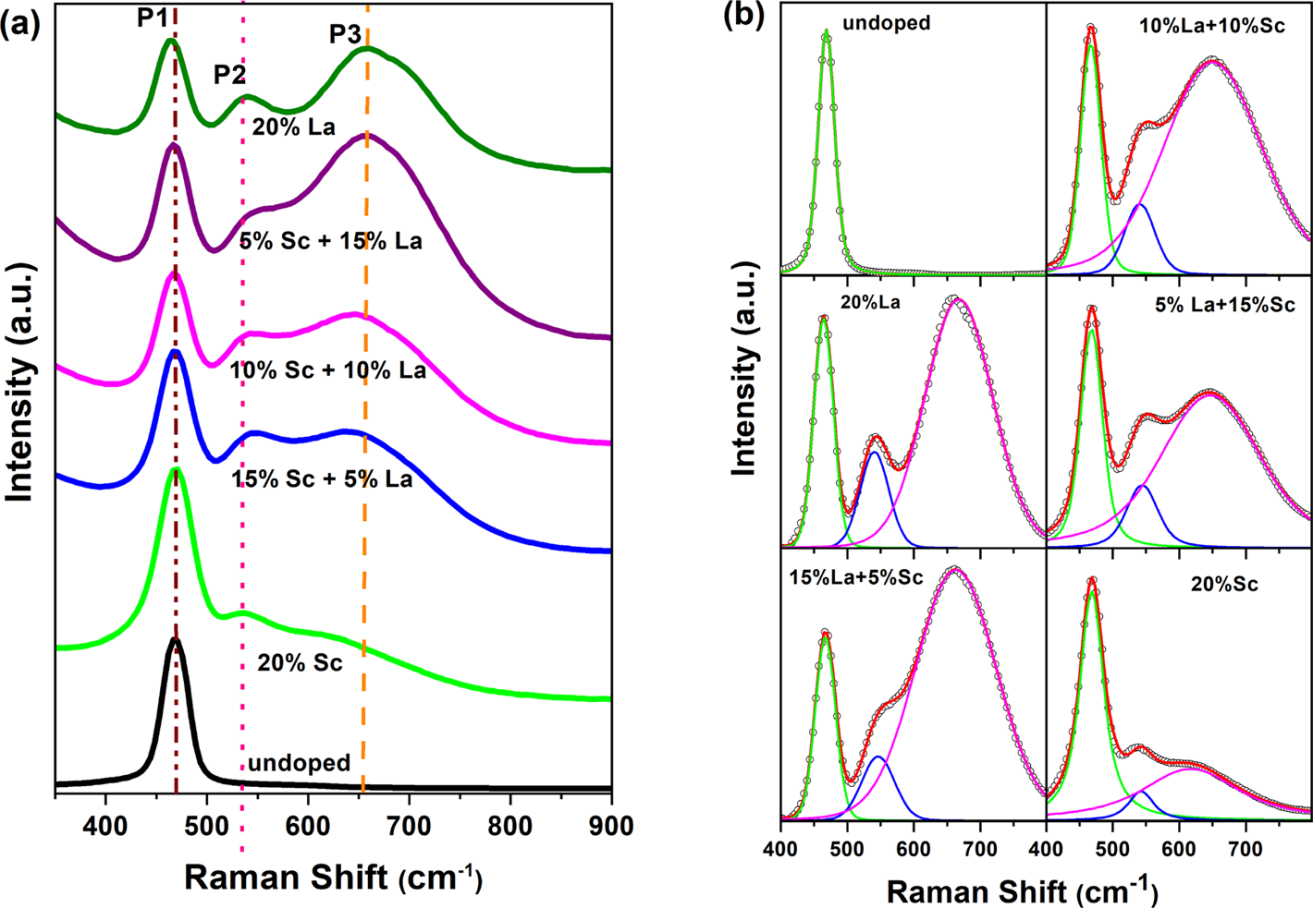
(a) Raman spectra and (b) deconvoluted Raman spectra of La- and Sc-doped CeO_2_.

Raman spectroscopy is another technique used to understand defects in trivalent-ion-doped samples. The Raman spectra of the doped samples are shown in Fig. 6a. Undoped ceria shows a single prominent peak at 469 cm^−1^ (denoted as P1), which can be attributed to the F_2g_ symmetry mode in the CeO_2_ lattice arising due to the symmetric breathing mode of the oxygen atoms around Ce^4+^.^42,43^ Upon doping with Sc and La, the F_2g_ band becomes broader and red-shifts (moves to a lower wavenumber) with an increase in the fraction dopant La. Similar broadening and shift in La-doped ceria with increasing doping levels has also been reported in earlier studies.^44^ The shift was observed to be higher in La- and Nd-doped samples compared with Eu- and Pr-doped ceria, though the size of Nd is less than Pr and more than Eu.^19^

**Fig. 7:**
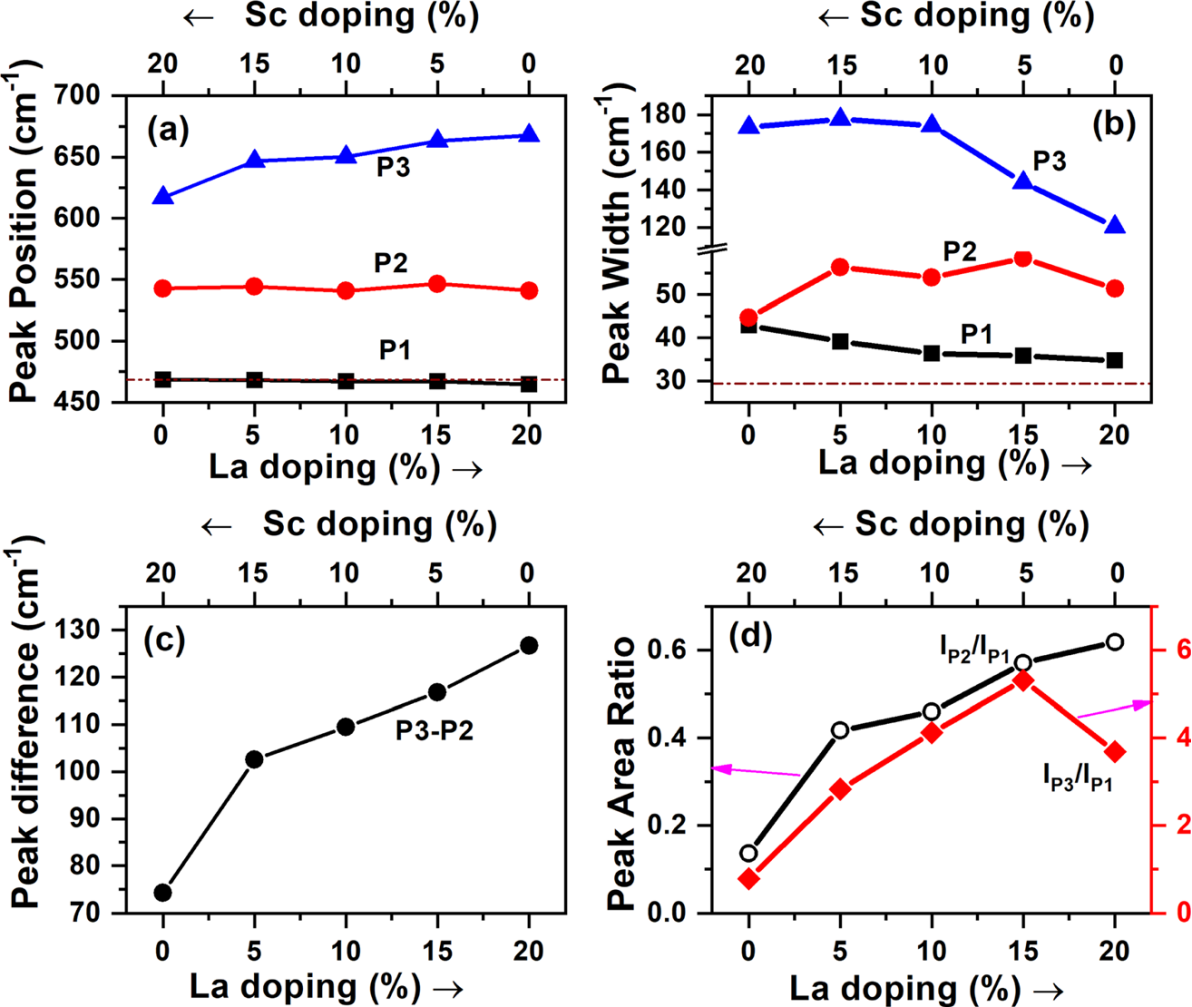
(a–d) Different parameters obtained from the deconvoluted Raman spectra shown in Fig. 6. The dotted lines in (a and b) correspond to the F_2g_ peak in undoped ceria.

In addition to the broader F_2g_ band, two other bands, one around 540–550 cm^−1^ and an even broader band around 625–675 cm^−1^, were also observed for all the samples. The defect bands at around 560 cm^−1^ (denoted as P2) were observed for all trivalent-ion-doped ceria but were absent in tetravalent-ion-doped ceria.^45^ The relative area under the peak at ∼560 cm^−1^ with respect to the F_2g_ band intensity can be taken as the relative concentration of oxygen vacancies. The band beyond 600 cm^−1^ (denoted as P3) has been attributed to Ce^3+^ in the CeO_2_ lattice, and its relative area with respect to F_2g_ can be used as the measure of Ce^3+^ in the doped samples.^42^ The same has also been attributed to the presence of dopant ions of different ionic radii and the distortions caused by them in the oxygen lattice positions.^44,46^ This peak was absent in undoped ceria though Ce^3+^ content is seen from XPS studies. This may mean that Ce^3+^ is less in the surface regions, where Raman is sensitive, or this peak is seen mainly due to distortions caused by the presence of ions of different ionic radii, as suggested by Chen *et al.*^46^ The Raman spectra demonstrating the shift in the region of 400–800 cm^−1^ have been deconvoluted and shown in Fig. 6b. The different parameters obtained from the deconvoluted spectra are shown in Fig. 7.

It is seen from Fig. 7 that the position of the F_2g_ peak is slightly redshifted with an increase in the La^3+^ dopant fraction besides peak broadening (Fig. 7a, P1), but the width lowers when the La^3+^ fraction is much higher (Fig. 7b, P1). The redshift of the F_2g_ Raman peak is reported whenever the lattice constant of ceria increases with the doping of larger trivalent ions than Ce^4+^ (ref. 19 and 44) or under temperature and pressure.^47^ Lesser redshifts with smaller dopants are also reported by Pikalova *et al.* using various trivalent dopants.^48^ The lesser width of the F_2g_ peak is attributed to better crystallinity and lower lattice strain in the samples, and the current data suggests that when the La^3+^ dopant fraction is higher, the strain is lower or crystallinity is better.^49,50^ The position of the peak of oxygen vacancy defect (P2) did not vary monotonically but was lower when an equal fraction of Sc^3+^ and La^3+^ was doped than individual doping. The width of this peak was higher, and the variation in width showed an opposite trend to that of F_2g_. The third band attributed to Ce^3+^-related vacancies was blue-shifted, and the width was reduced with an increase in La^3+^ dopant fraction; consequently, the difference in peak positions of P2 and P3 increased with increasing La^3+^ fraction. The ratios of the areas (intensities) under the defect peaks with respect to the F_2g_ peak area are shown in Fig. 7d. The ratio of the peak corresponding to oxygen vacancies increased monotonically with the La^3+^ fraction. From the photoluminescence studies, which show a weak association of oxygen vacancies with La^3+^ and is also supported by theoretical studies in the literature, it can be concluded that in the samples with a higher Sc^3+^ fraction, the oxygen vacancies are associated with Sc and are distributed more in the bulk, while they are more on the surface when the La^3+^ fraction is high. The Ce^3+^ fraction (*I*_P3_/*I*_P1_) increased with the La fraction and reached the maximum in the 5% Sc + 15% La-doped sample but was found to be lower in the pure La-doped sample.

With an increase in the fraction of larger trivalent ions (La^3+^), the oxygen vacancies created are weakly associated with the dopant and aid Ce^3+^ formation. However, the ionic radius of Ce^3+^ is larger, and doping a larger trivalent ion may not favour reduction. The competing effects seem to have resulted in the higher concentration of Ce^3+^ in the sample codoped with La^3+^ and Sc^3+^, which have ionic radii that lie on either side of Ce^4+^. The positron annihilation lifetime spectroscopy, XPS, photoluminescence and Raman studies suggest that the mix and match of dopants with different ionic radii may be another option for tuning the oxygen vacancy concentration and distribution in ceria. It may also be noted that besides the changing relative fractions of the smaller and larger trivalent dopants, the concentration of the dopants is another factor that requires further attention.

### 3.7. Electrochemical studies

**Fig. 8:**
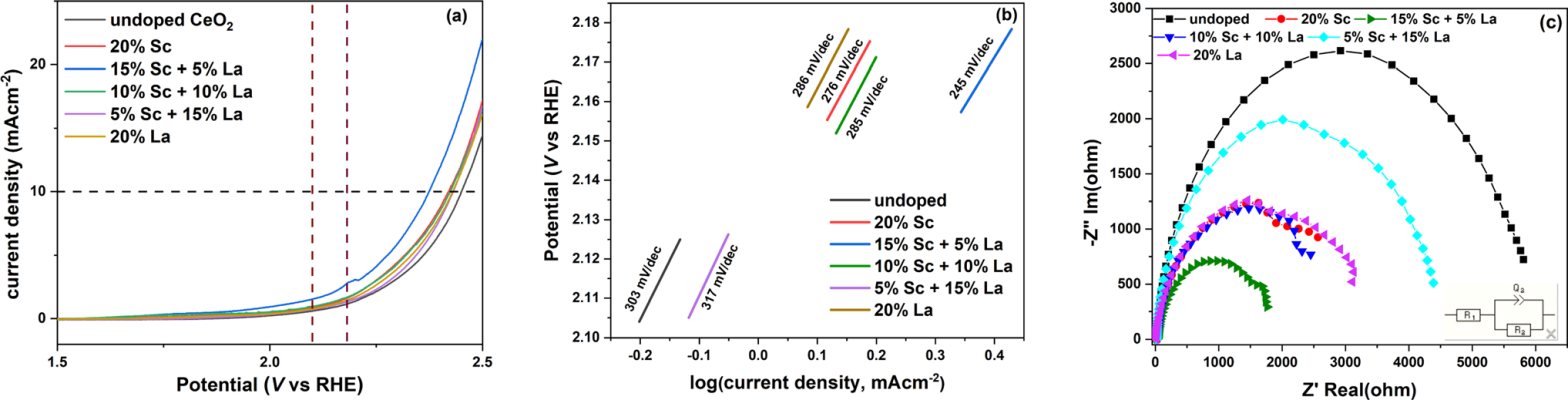
(a) Linear sweep voltammograms and (b) the corresponding Tafel plots (within the marked region) of the ceria catalysts for the OER in 1 M KOH solution. (c) Nyquist impedance plots of the various ceria catalysts for the OER. The inset figure shows the equivalent circuit used for fitting the impedance spectra.

The linear sweep voltammograms of the catalyst-modified GC electrodes used for OER in a 1 M KOH medium are shown in Fig. 8a. From the figure, it is clear that all the doped ceria catalysts showed superior catalytic activity in terms of current density compared with undoped ceria. Out of these, the catalyst codoped with 15% Sc and 5% La showed the highest current density. The oxidation reaction overpotential, which arises from various factors like activation barrier, concentration polarisation and ohmic drop, was calculated for all the catalysts at 10 mA cm^−2^ current density with respect to the thermodynamic potential of 1.23 V. The calculated values are listed in Table 1, and it is evident that aliovalent doping successfully lowered the overpotential of the OER process. Similar to the trend of the current density, the catalyst with 15% Sc and 5% La loading also showed the lowest overpotential of 1144 mV. The spectroscopic studies discussed above reveal that the Ce^3+^ fraction at the surface was the highest in the 15% Sc + 5% La codoped catalyst along with a substantial surface oxygen vacancy concentration and stronger association of the vacancies with Sc. Hence, it can be predicted that the synergistic effect of these factors may subsequently tune the covalency of the metal–oxygen (Ce–O) bond and facilitate oxygen evolution through a lattice oxygen evolution mechanism (LOM).^51^

**Table 1:** Calculated overpotential, double-layer capacitance and charge transfer resistance of the ceria catalysts for the OER

Dopant (s)	Overpotential (mV)	*Q*_1_ (μF)	*a*_1_	*R*_ct_ (ohm)
Undoped CeO_2_	1220	3.01	0.88	6079
20% Sc	1189	5.22	0.92	2342
15% Sc + 5% La	1144	11.36	0.80	1930
10% Sc + 10% La	1194	4.15	0.85	2912
5% Sc + 15% La	1200	3.74	0.95	4318
20% La	1199	5.48	0.93	2943

To further elucidate the crucial role of the aliovalent dopants on the kinetics and mechanism of the OER process, the Tafel plots of the catalysts were examined. The region selected for calculating the Tafel slope is marked by vertical lines in Fig. 8a based on the range of potential near the onset of the OER process. From the calculated values of Tafel slopes depicted in Fig. 8b, it is evident that the OER process is highly facile on the 15% Sc + 5% La codoped catalyst as it shows a substantially lower Tafel slope of 245 mV per decade than that of undoped ceria. The Tafel slope values are quite comparable to those reported in other aliovalent-ion-doped ceria catalysts.^16^ However, the catalyst codoped with 15% La and 5% Sc showed significantly slower kinetics with a relatively higher value of Tafel slope, which is contradictory to the response observed in terms of overpotential and current density. The possible reason for this anomaly is the presence of a greater number of larger defect traps, as reflected by the highest average defect density, as well as the preferential distribution of Ce^3+^ in the bulk, for this particular dopant composition.

To further gain insights into the charge transfer process, electrochemical impedance spectroscopy (EIS) was carried out, and the fitted Nyquist impedance plots are shown in Fig. 8c. The equivalent circuit used for analysing the spectra is shown in the inset, which is similar to the simplest Randles circuit, one of the most common equivalent circuits used for metal-oxide electrodes. In this circuit, *R*_1_ denotes *R*_sol_, which refers to the resistance of the electrolyte, connectors, wires and leads between the reference and working electrodes. *Q*_1_ denotes a constant phase element, which signifies the double layer capacitance at the electrode–electrolyte interface here. *R*_2_ represents the faradaic electron transfer or charge transfer resistance towards the OER process.^52^ The equivalent circuit not only takes into account the changes occurring at the working electrode–electrolyte interface but also the contribution of the solution resistance between the reference and working electrodes and effectively represents our experimentally observed results. The value of *R*_sol_ was evaluated to be around 26 ohms, and the fitted values of *R*_ct_ and double-layer capacitance are listed in Table 1. From Table 1, it is quite evident that the 15% Sc and 5% La-doped catalyst not only shows the best response in LSV and Tafel measurements but also exhibits an appreciably low resistance towards the charge transfer phenomenon during the oxidation process. This lowering of *R*_ct_ can be attributed to the faster electron flow through the catalyst surface *via* hydroxyl ions.^16^ Interestingly, it may be further noted here that this particular codoped catalyst has the closest match of effective ionic radii between the dopants and the host. To further check the stability of the best-performing 15% Sc and 5% La codoped catalyst, a chronopotentiometric test was carried out, and the catalyst exhibited excellent stability, as depicted in Fig. S6 (ESI†).

Therefore, it can be summarised that effective ionic radius modulation using a combination of dopants can be a smart strategy to introduce optimum defects and tune the surface Ce^3+^ concentration, which altogether can influence and improve the catalytic activity of metal-oxide-based catalysts. Moreover, such tuning of the effective ionic radii *via* aliovalent doping of metals with higher conductivity can be further advantageous in this regard.^38,53^

## 4. Conclusions

Defect engineering *via* aliovalent doping was carried out in ceria catalysts to improve the electrocatalytic activity in water-splitting reactions. Effective ionic radii modulation was achieved by the incorporation of a smaller (Sc^3+^) and a larger dopant (La^3+^), and the ceria catalysts were also spiked with Eu^3+^ to utilize its emission features for understanding the association between defects and dopants. The catalysts were synthesized using a gel-combustion method, and doping was confirmed by XRD, Rietveld refinement and neutron activation analysis. The combined effect of defect evolution and Ce^3+^ content on the properties of the doped ceria catalysts were investigated by Positron Annihilation Lifetime Spectroscopy (PALS), X-ray Photoelectron Spectroscopy (XPS), Photoluminescence (PL) spectroscopy and Raman spectroscopy. Electrochemical studies were performed to explore their efficacy in catalysing OER in alkaline medium. These studies indicate that the catalyst with optimum defect density, the maximum Ce^3+^ fraction at the surface of the catalyst, and the least difference in effective ionic radii between the dopants and the host exhibited exceptional performance towards OER. These findings pave the way for designing efficient defect-engineered ceria catalysts based on the rational choice of co-dopants, cheap and effective synthesis strategy and defect concentration tuning.

## Conflicts of interest

There are no conflicts to declare.

## Supplementary Material

RA-014-D4RA03360D-s001
