# Supplementary material for: Modulating the effective ionic radii of trivalent dopants in ceria using a combination of dopants to improve catalytic efficiency for the oxygen evolution reaction
Source: RSC Adv. 2024 Jun 3;14(25):17801–13. doi: 10.1039/d4ra03360d (PMC11145625; doi:10.1039/d4ra03360d)
Supplement: RA-014-D4RA03360D-s001 [file RA-014-D4RA03360D-s001.pdf]

Electronic Supplementary Material (ESI) for RSC Advances.
This journal is © The Royal Society of Chemistry 2024

Modulating the Effective Ionic Radii of Trivalent dopants in Ceria by Combination of Dopants for Improving Catalytic Efficiency towards Water Splitting Reactions

Debarati Das,[1,2] Jyoti Prakash, [2,3] Anisha Bandyopadhyay,[2,4] Annu Balhara,[1,2] U.K. Goutam,[5] Raghunath Acharya,[1,2] Santosh K. Gupta,[1,2,*] and Kathi Sudarshan[1,2,#]

[1]Radiochemistry Division, Bhabha Atomic Research Centre, Mumbai-400085, India

[2]Homi Bhabha National Institute, Anushaktinagar, Mumbai – 400094, India

[3] Materials Science Group, Bhabha Atomic Research Centre, Mumbai-400085, India

[4]Radiation and Photochemistry Division, Bhabha Atomic Research Centre, Mumbai-400085, India

[5]Technical Physics Division, Bhabha Atomic Research Centre, Mumbai-400085, India

S1. Rietveld Refinement and Williamson-Hall Analysis:

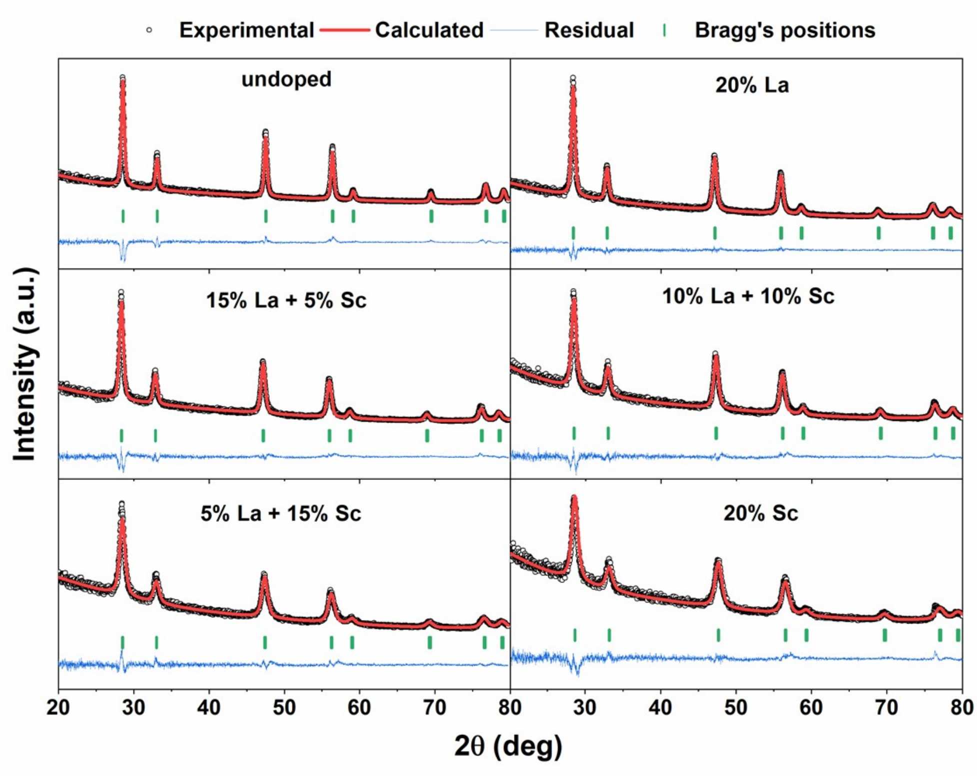

Figure S1: Rietveld refined XRD patterns of the undoped, doped and codoped CeO_2 nano-powders

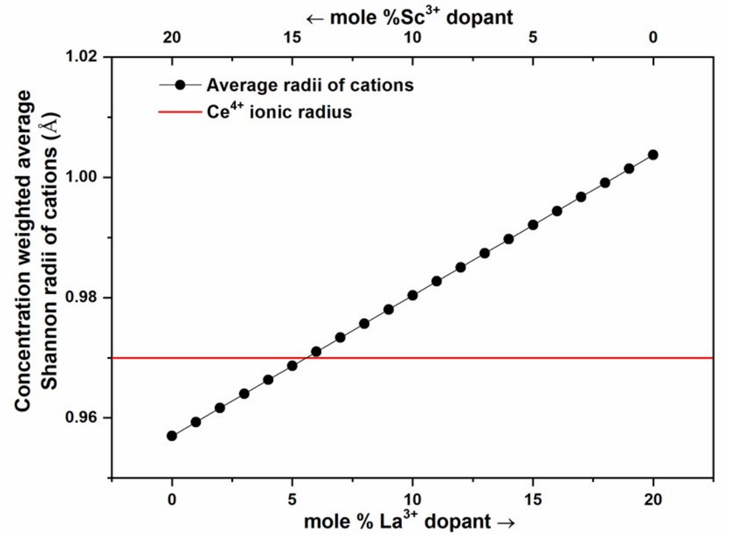

Figure S2: Concentration weighted Shannon radii of cations in the doped ceria samples.

S1.1. Methodology of Williamson-Hall analysis:

In Williamson-Hall approach, based on uniform deformation model, broadening of the X-ray diffraction peak is calculated considering independent contribution from crystallite size and lattice strain using the following equation.

$$\beta_{hkl}cos\theta_{hkl} = \frac{K\lambda}{D} + 4\varepsilon sin\theta_{hkl}$$

Where, $\beta_{hkl}$is expressed as: $\sqrt{(\beta_{sample}{}^2 - \beta_{instr}{}^2)}$, the instrumental broadening corrected FWHM (in radians) of a given XRD peak fitted using a gaussian profile, K is a constant equal to 0.9, wavelength λ for Cu K_α is 1.5406 Å, D is crystallite size and ε is themicro-strain. Instrumental broadening is calculated from the selected peak widths of the XRD pattern of LaB_6standard. Strain and crystallite size were calculated respectively from the slope and intercept of the linear plot of $\beta_{hkl}cos\theta_{hkl}$vs ,$4sin\theta_{hkl}$.

Table S1: Rietveld refinement fitting parameters and estimated crystallite size of the undoped, doped and codoped CeO_2 catalysts:

(Number in the parenthesis denotes the uncertainty in the least significant digit)

Dopant(s)	Rietveld refined lattice parameter (Å)	Unite cell volume (Å^3)	Goodness of Rietveld fitting parameters	Crystallite size from WH analysis (nm)
Undoped	5.412 (1)	158.49(1)	χ^2 = 3.39<br>R_p = 5.95<br>R_{wp}= 8.35<br>R_{exp}= 4.54	30

20% Sc	5.397(1)	157.24(2)	$\chi^2 = 2.04$<br>$R_p = 4.67$<br>$R_{wp} = 6.31$<br>$R_{exp} = 4.42$	13
15% Sc + 5% La	5.425(1)	159.70(1)	$\chi^2 = 1.65$<br>$R_p = 4.16$<br>$R_{wp} = 5.45$<br>$R_{exp} = 4.25$	19
10% Sc + 10% La	5.435(1)	160.58(2)	$\chi^2 = 2.05$<br>$R_p = 4.52$<br>$R_{wp} = 6.11$<br>$R_{exp} = 4.35$	16
5% Sc + 15% La	5.448(1)	161.73(2)	$\chi^2 = 2.62$<br>$R_p = 5.29$<br>$R_{wp} = 7.43$<br>$R_{exp} = 4.41$	21
20% La	5.455(1)	162.29(1)	$\chi^2 = 1.43$<br>$R_p = 3.97$<br>$R_{wp} = 5.11$<br>$R_{exp} = 4.28$	25

S2. Neutron Activation Analysis and TEM morphological studies:

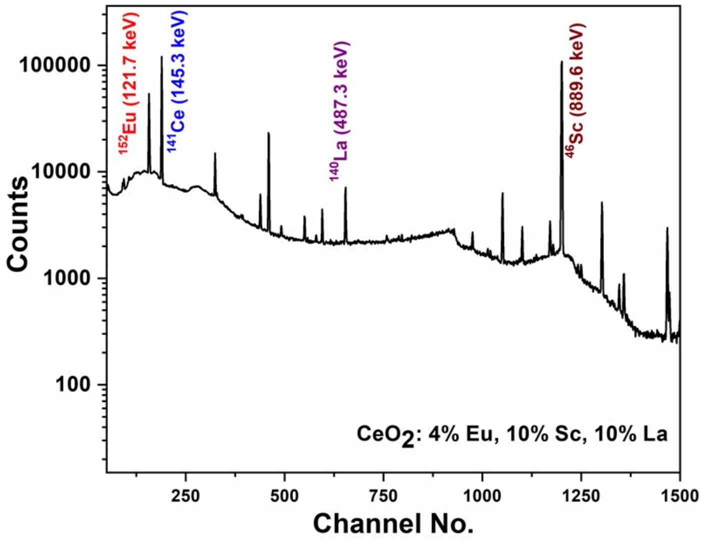

Figure S3: Typical gamma ray spectrum of neutron irradiated Sc, La codoped CeO_2 spiked with 4% Eu.

S3. Positron Annihilation Lifetime Spectroscopy:

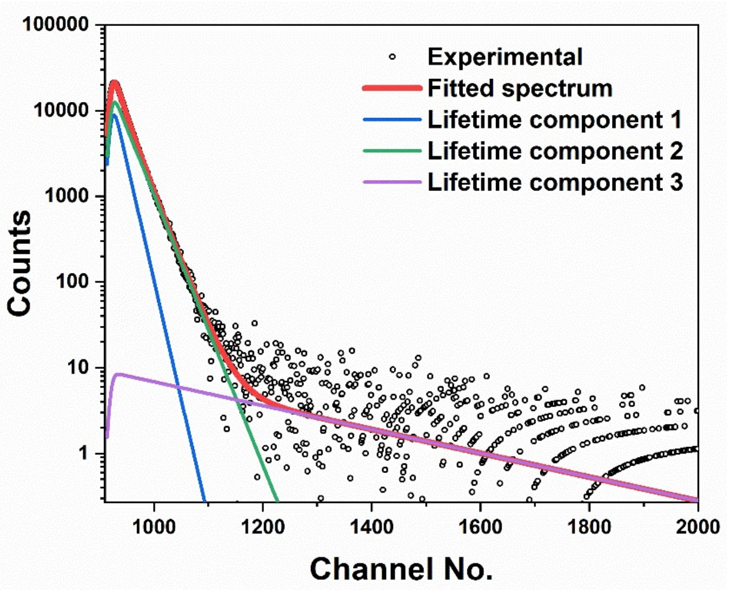

Figure S4: A typical fitted positron lifetime spectrum of 10% Sc + 10% La codoped ceria catalyst

S4. X-ray photoelectron spectroscopy:

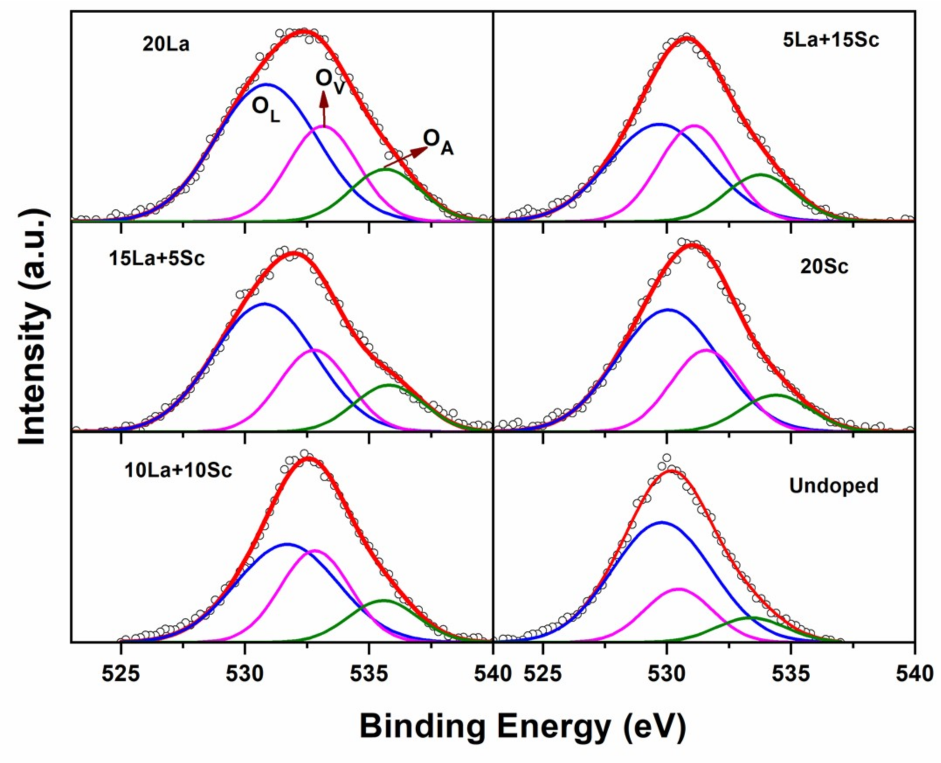

Figure S5: Fitting of O-1s XPS spectra of the ceria catalysts

S5. Electrochemical Studies:

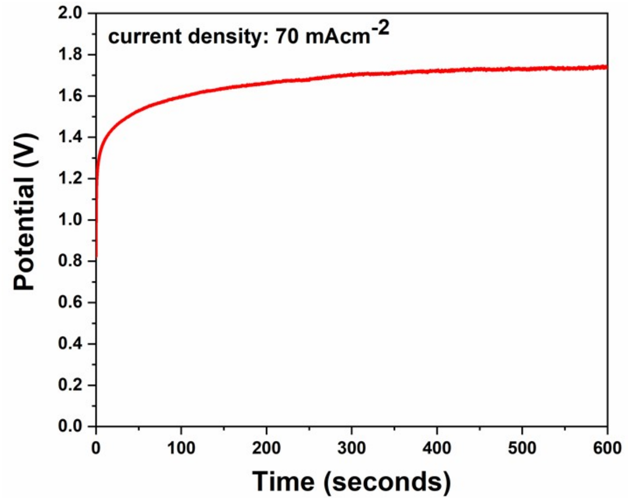

Figure S6: Chronopotentiometric stability test of the best performing 15% Sc+ 5% La doped catalyst towards OER.
